# Global Survey, Expressions and Association Analysis of *CBLL* Genes in Peanut

**DOI:** 10.3389/fgene.2022.821163

**Published:** 2022-03-09

**Authors:** Weifang Ren, Zhaocong Zeng, Sijian Wang, Juncheng Zhang, Jiahai Fang, Liyun Wan

**Affiliations:** ^1^ Key Laboratory of Crop Physiology, Ecology and Genetic Breeding, Ministry of Education, Jiangxi Agricultural University, Nanchang, China; ^2^ Southern Regional Collaborative Innovation Center for Grain and Oil Crops in China, Nanchang, China; ^3^ College of Agronomy, Jiangxi Agricultural University, Nanchang, China; ^4^ Huazhong Agricultural University, Wuhan, China

**Keywords:** peanut, cis-acting element, genome-wide association analysis, CBLL gene family, expression pattern

## Abstract

Cystathionine γ-synthase (CGS), methionine γ-lyase (MGL), cystathionine β-lyase (CBL) and cystathionine γ-lyase (CGL) share the Cys_Met_Meta_PP domain and play important roles in plant stress response and development. In this study, we defined the genes containing the Cys_Met_Meta_PP domain (PF01053.20) as *CBL-*like genes (*CBLL*). Twenty-nine *CBLL* genes were identified in the peanut genome, including 12 from cultivated peanut and 17 from wild species. These genes were distributed unevenly at the ends of different chromosomes. Evolution, gene structure, and motif analysis revealed that *CBLL* proteins were composed of five different evolutionary branches. Chromosome distribution pattern and synteny analysis strongly indicated that whole-genome duplication (allopolyploidization) contributed to the expansion of *CBLL* genes. Comparative genomics analysis showed that there were three common collinear *CBLL* gene pairs among peanut, Arabidopsis, grape, and soybean, but no collinear *CBLL* gene pairs between peanut and rice. The prediction results of *cis*-acting elements showed that *AhCBLLs*, *AdCBLLs, and AiCBLLs* contained different proportions of plant growth, abiotic stress, plant hormones, and light response elements. Spatial expression profiles revealed that almost all *AhCBLLs* had significantly higher expression in pods and seeds. All *AhCBLL*s could respond to heat stress, and some of them could be rapidly induced by cold, salt, submergence, heat and drought stress. Furthermore, one polymorphic site in *AiCBLL7* was identified by association analysis which was closely associated with pod length (PL), pod width (PW), hundred pod weight (HPW) and hundred seed weight (HSW). The results of this study provide a foundation for further research on the function of the *CBLL* gene family in peanut.

## Introduction

Sulfur-containing amino acids play an important role in the growth and development of plants and animals (Lorraine et al., 1986). When plants lack sulfur-containing amino acid, their metabolic processes will be abnormal and growth will be affected (Kery et al., 1994). Methionine (Met) is a sulfur-containing amino acid that is essential to all organisms and indirectly regulates a variety of cellular processes through S-adenosine methionine (SAM) (Amir, 2010). SAM is also a methyl donor for the methylation of proteins, lipids, DNA, and RNA, and is a precursor to biosynthesis of the plant hormones ethylene, polyamines, and biotin (Benkova et al., 2003). A recent study showed that Met activates GLR (glutamate receptor), thereby activating Ca^2+^ channels that regulate stomatal movement and plant growth (Galili et al., 2016). Met also promotes root morphogenesis and enhances chlorophyll content to enhance photosynthesis (Sarropoulou et al., 2013).

Met biosynthesis requires cystathionine γ-synthase (CGS), cystathionine β-lyase (CBL), and Met synthase enzymes (Messerschmidt et al., 2003). In plants, CGS first converts cystine (Cys) and activates the conversion of homoserine to cystathionine. Cystathionine is then cleaved by CBL to yield homoCys, pyruvate, and ammonia. Met synthase finally methylates homo-Cys to produce Met (Ravanel et al., 1998). Animals and some microorganisms can metabolize Met back to Cys by a reverse trans-sulfuration pathway that involves cystathionine β-synthase (CBS) and cystathionine γ-lyase (CGL). Plants and certain bacteria are proposed to have an alternative route (or routes) to convert methionine to cysteine, of which the first step is mediated by methionine γ-lyase (MGL). The subsequent steps in this pathway(s) have not been definitively established (Goyer et al., 2007). Arabidopsis mutants perturbed in Met metabolism have been described,*mto* alleles carried single base-pair mutation in a conserved domain led to over-accumulation of Met (Goto et al., 2002). Over-expression of *AtCGS* caused an increased level of Met, which induced the up-regulation of genes involved in ethylene and abscisic acid homeostasis and light, sucrose, salt and osmotic stresses regulation (Hacham et al., 2013; Cohen et al., 2014, 2017; Whitcomb et al., 2018). Pharmacological treatment of plants with inhibitors against *CGS* or *CBL* induced a deficiency in Met biosynthesis and caused growth inhibition (Ravanel et al., 1998). Knockdown of *CGS* and *CBL* in Arabidopsis led to abnormal leaf development stunted (Kim and Leustek., 2000; Levin et al., 2000). CBL is crucial for embryo patterning and the maintenance of the root stem cell niche in Arabidopsis (Liu et al., 2019). The Met homeostasis gene *METHIONINE GAMMA LYASE* (*AtMGL*) is up-regulated by dual stress in leaves, conferring resistance to nematodes when overexpressed, *AtMGL* regulates Met metabolism under conditions of multiple stressors, Met degradation, and plays a subordinate role to threonine deaminase (Goyer et al., 2007; Joshi and , 2009; Atkinson et al., 2013). A similar strategy has been applied to produce transgenic plants with reduced *CBL* levels in potato. These *CBL* antisense plants exhibited a short bushy stature, altered leaf morphology, and small tuber size (Maimann et al., 2000). In soybean, the overexpress of *AtD-CGS* notably increased the level of soluble Met in developing green seeds (3.8–7-fold), and these soybean seeds also showed high levels of other amino acids; furthermore, the total Met content, which included Met incorporated into proteins, notably increased in the mature dry seeds of these two transgenic lines by 1.8- and 2.3-fold, respectively (Song et al., 2013).

Peanut is an annual leguminous herb, is widely cultivated around the world as an important source of oil and protein for humans (Yang et al., 2014; Liao et al., 2018; Bertioli et al., 2019). Peanut kernels are rich in natural nutrients such as proteins, fatty acids (FAs), vitamins, minerals, and fiber (Chen et al., 2010). Studies have demostrated that the protein content of peanut seeds is around 23–33%; however, the content of sulfur-containing Met and Cy is low, especially for the former (Yang et al., 2001). Met and the enzyme coding genes involved in the Met/Cys interconversion pathway play inhibitory roles in both amino acid composition and content of storage protein, and also in tissue development and stress response regulation. Therefore, it is of great significance to further explore the *CBLL* gene family in peanut and identifythe function of family members in improving peanut yield, quality, and stress tolerance. In this study, we defined the genes containing the Cys_Met_Meta_PP domain (PF01053.20) as *CBL-*like genes (*CBLL*). Twenty-nine *CBLL* genes were identified from the peanut genome, whose conserved domains, phylogenetic tree, chromosome distribution, gene structure and expression pattern were analyzed. The results provided a basis for the role of peanut *CBLL* in the development and formation of peanut pods, and stress response regulation. This research laid a foundation for the identification and utilization of peanut *CBLL* genes, which is of great significance for the molecular based breeding of cultivars with multi-resistance, high yield, and good quality.

## Materials and Methods

### Identification of the *CBLL* Genes in Peanut

To identify *CBLL* genes in peanut, the predicted protein sequences were downloaded from PeanutBase (https://peanutbase.org) (*Arachis Duranensis* V14167: A-genome; *Arachis Ipaensis* K30076: B-genome). The Cys_Met_Meta_PP domain (PF01053.20) was identified from the protein sequences by using the HMMER 3.0 program at a standard E-value < 1 × 10^−5^ (Deng et al., 2019; Misra et al., 2019). Conserved domain searches were performed against the conserved domain database in NCBI (http://www.ncbi.nlm.nih.gov/Structure/cdd/wrpsb.cgi). Members with incomplete conserved functional domains were removed. The *CBLL* genes were named as *AdCBLL1* to *AdCBLL9*, *AiCBLL1* to *AiCBLL8*, and *AhCBLL1* to *AhCBLL12* according to their positions on peanut chromosomes. Physicochemical parameters of peanut CBLL proteins were then generated by ProtParam Tools, including theoretical isoelectric points (pI) and molecular weights (MW) (Gasteiger et al., 2005).

### Analyses of Phylogeny, Gene Structure, and Conserved Motifs

Phylogenetic analysis was carried out based on the protein sequences of the *CBLL* genes in peanut (*A.hypogaea* L., *A.duranensis, A.ipaensis*) and Arabidopsis. The protein sequences were aligned by ClustalW, and the unrooted Neighbor-Joining (NJ) phylogenetic tree was constructed by MEGA 5.2 software with 1,000 bootstrap replicates. Gene annotation information was downloaded from PeanutBase (http://www.peanutbase.org/) and GSDS 2.0 (http://gsds.gao-lab.org/) was used to visualize the gene structure. The composition of conserved motifs was searched by the Multiple EM for Motif Elicitation (MEME) online tool by setting a maximum number as 20 (http://meme-suite.org/tools/meme).

### Gene Duplication and Synteny Analysis of the Peanut *CBLL* Genes

MCScan (http://chibba.agtec.uga.edu/duplication/mcscan) was used to identify *AhCBLs* duplications and the synteny block of *CBLL* genes of peanut and the other four species (Arabidopsis, rice, grape and soybean). BLASTP was applied to find homologous sequences of *CBLL* genes between peanut and Arabidopsis, after that literatures were reviewed to explore the function of published *CBLL* genes in Arabidopsis. Tandem duplications were defined as adjacent homologous genes on the same chromosome with a distance of <50 kb (Cannon et al., 2004). If they were paralogs located on duplicated chromosomal blocks, they were defined as a segmental duplication event (Guo et al., 2016). Non-synonymous (Ka) and synonymous (Ks) substitution of each duplicated genes were calculated using the PAL2NAL program (Suyama et al., 2006), which was based on the codon model program in PAML (Yang, 2007).

### *Cis*-Acting Element Analysis in the Promoters of Peanut *CBLLs*

Regulatory elements of promoter sequences can control gene expression. The 2-kb promoter sequences of 29 *CBLL* genes were downloaded from PeanutBase (http://www.peanutbase.org/) and used to predict the *cis*-regulatory element through the PlantCARE database (http://bioinformatics.psb.ugent.be/webtools/plantcare/html/) (Lescot, 2002). The radar figures were manually generated by R3.5.1 scripts.

### Expression Profiles of *AhCBLL* Genes in Different Tissues

RNA-seq data sets of 22 peanut tissues were downloaded from PeanutBase and the NCBI SRA database to explore the expression profiles of *AhCBLL* genes in different tissues, which were submitted by Clevenger et al. (Clevenger et al., 2016).

### Plant Materials, Growth Conditions and Treatments

Different experimental treatments were carried out for 10-day-old seedlings of a Chinese elite peanut cultivar Changhua18 which were planted in vermiculite and irrigated with sterilized water (26°C, 16-h light/8-h dark). For hormone treatments, seedlings were sprayed with solutions containing 6-benzyl amino purine (6-BA) (25 μM), indole-3-acetic acid (IAA) (50 μM), gibberellic acid (GA) (100 μM), salicylic acid (SA) (100 μM), abscisic acid (ABA) (100 μM), ethylene (ACC) (500 μM) and methyl jasmonate (MJ) (100 µM)(Jain et al., 2006; Wang et al., 2016). For heat and cold stresses, the 10-day-old seedlings were transferred to two environmental temperatures of 40°C (H40) and 4°C (Cold 4) respectively (Jain et al., 2006; Wang et al., 2016). Seedling samples were collected at 0, 1, 3, 6, 9 and 12 h after the above treatment. For submergence (Sub), the seedlings were soak in water to a depth of 5 cm from tip to surface, and samples were collected at 0, 6, 12, 24, 48, and 72 h after treatment (Wang et al., 2016). For the NaCl and polyethylene glycol (PEG) treatments, the 10-day-old seedlings were immersed in NaCl solution (200 mM) and PEG6000 (20%, w/v) (Jain et al., 2006; Song et al., 2009), and samples were collected after 0, 0.5, 1, 3, 6, 12, and 24 h after seedling treatment. Three biological replicates were performed; each sample included around eight seedlings. All samples were placed in liquid nitrogen during sampling and stored at −80°C to preserve RNA integrity.

The genotype data of the *CBLL* genes used here were obtained from transcriptome sequencing data of a peanut germplasm population with 146 accessions (unpublished data). Each line of the peanut populations was planted in five different environments (Wuhan 2016, Wuhan 2017, Yangluo 2016, Yangluo 2017, and Zhanjiang 2016). All seedlings were planted within the experimental plot with 12 plants in a line in each environment.

### RNA Isolation and qRT-PCR Analysis

Total RNA of samples were isolated using TRIzol reagent (Invitrogen) according to the manufacture’s requirements. M-MLV reverse transcriptase (Promega) was used to synthesize the first chain of cDNA from 5 µg total RNA. Quantitative Real-Time PCR (qRT-PCR) was performed using 2×SYBR Green Master Mix (Bio-Rad) on a 96-well plate with a gene-specific primer (Supplementary Table S1). The thermal cycle was as follows: 95°C for 5 min; 40 cycles of 95°C for 10 s, primer-specific annealing temperature of, 72°C for 10 s, for 15 cycles; then the melt curve was from 65 to 95°C.

## Results

### Identification of the *CBLL* Gene Family in Peanut

In order to identify *CBLL* gene families in peanut, we downloaded the published peanut genome sequence from PeanutBase (https://peanutbase.org/). The Cys_Met_Meta_PP domain (PF01053.20) containing proteins were identified by HMMER 3.0 with a standard E-value < 1 × 10^−5^; further, we removed the incomplete sequences, and identified 29 *AhCBLL* members (Table 1) from the cultivated peanut (*A. hypogaea* L.) and its diploid progenitors (*A. duranensis*, *A. ipaensis*). Table 1 summarizes genes with complete sequences, among which 17 *CBLLs* were from A-genome, and 12 *CBLLs* were from B-genome; they were distributed unevenly across chromosomes. The 12 members from *A. hypogaea* L. were named as *AhCBLL1∼AhCBLL12*, the nine *AhCBLLs* from *A.duranensis* were named as *AdCBLL1∼AdCBLL9*, and the eight *AhCBLLs* from *A.ipaensis* were named as *AiCBLL2∼AiCBLL8*, respectively, according to their chromosomal order. We then determined the chromosome location, amino acid number (AA), mRNA length, theoretical isoelectric points (pI) and other information of peanut CBLLs (Table 1). The open reading frame (ORF) lengths of the *CBLL* genes ranged from 462 bps to 5,944 bps (Table 1). The protein sequences of the peanut *CBLL* genes were significantly different; sequence lengths ranged from 77 to 1855 aa. The molecular weights (MWs) of AdCBLLs varied from 8.44 kDa (AdCBLL3) to 53.51 kDa (AdCBLL5); AiCBLLs ranged from 15.03 kDa (AiCBLL6) to 60.63 kDa (AiCBLL8); and AhCBLLs varied from 15.69 kDa (AhCBLL8) to 205.59 kDa (AhCBLL5). The pI was small for the overwhelming majority of CBLLs, ranging from 4.92 (AhCBLL3) to 9.07 (AiCBLL1). AhCBLL8 carried one conservative transmembrane domain (TMDs), AdCBLL4 carried two TMDs, and other peanut CBLLs did not contain TMDs.

**TABLE 1:** *CBLL* genes identified in peanuts.

Gene name	Gene locus	CDS length (bp)	AA^a^	MW (kDa)^b^	pI^c^	TMD^d^	Chr
*AdCBLL1*	*Aradu.02IGP*	1,520	426	46.17	5.93	0	Aradu.A04
*AdCBLL2*	*Aradu.M0JX8*	1,158	248	27.97	8.77	0	Aradu.A04
*AdCBLL3*	*Aradu.V9ADB*	618	77	8.44	7.82	0	Aradu.A04
*AdCBLL4*	*Aradu.N0LVM*	772	182	20.62	5.01	2	Aradu.A04
*AdCBLL5*	*Aradu.W013I*	1,545	497	53.51	6.02	0	Aradu.A04
*AdCBLL6*	*Aradu.JQ7JG*	1,843	464	50.42	6.18	0	Aradu.A06
*AdCBLL7*	*Aradu.93LBV*	1,020	208	22.83	4.96	0	Aradu.A06
*AdCBLL8*	*Aradu.UE7BN*	1,758	308	33.45	6.30	0	Aradu.A10
*AdCBLL9*	*Aradu.FE0Z7*	1,441	313	35.17	6.76	0	None
*AiCBLL1*	*Araip.3V2A7*	946	204	22.36	9.07	0	Araip.B01
*AiCBLL2*	*Araip.V3B0A*	1,654	426	46.07	6.01	0	Araip.B04
*AiCBLL3*	*Araip.I7QC8*	1,891	137	15.40	6.09	0	Araip.B04
*AiCBLL4*	*Araip.26T6F*	1,635	471	51.31	7.64	0	Araip.B04
*AiCBLL5*	*Araip.P8SRT*	1,858	464	50.39	6.06	0	Araip.B06
*AiCBLL6*	*Araip.B0SMD*	2,090	141	15.03	5.28	0	Araip.B09
*AiCBLL7*	*Araip.H1TPN*	1,724	442	48.10	6.25	0	Araip.B09
*AiCBLL8*	*Araip.KUG1C*	2,237	564	60.63	5.90	0	Araip.B10
*AhCBLL1*	*Arahy.AA870A*	1,876	250	26.57	5.30	0	Arahy.04
*AhCBLL2*	*Arahy.C0PD3X*	462	153	16.85	8.45	0	Arahy.04
*AhCBLL3*	*Arahy.2E7M4N*	623	204	22.79	4.92	0	Arahy.04
*AhCBLL4*	*Arahy.0JH0K6*	1,822	531	56.85	6.40	0	Arahy.04
*AhCBLL5*	*Arahy.F5KNV4*	5,885	1,855	205.59	6.00	0	Arahy.06
*AhCBLL6*	*Arahy.AGM2GS*	2,887	560	60.28	5.85	0	Arahy.10
*AhCBLL7*	*Arahy.V9A8GQ*	1,852	426	46.07	6.01	0	Arahy.14
*AhCBLL8*	*Arahy.Q4SW2C*	915	139	15.69	8.13	1	Arahy.14
*AhCBLL9*	*Arahy.I2BVW6*	1,815	531	56.81	6.40	0	Arahy.14
*AhCBLL10*	*Arahy.0AC10P*	5,944	1,830	202.91	5.98	0	Arahy.16
*AhCBLL11*	*Arahy.1FPN7V*	1,646	442	48.11	6.25	0	Arahy.19
*AhCBLL12*	*Arahy.S7AN2D*	2,691	564	60.63	5.90	0	Arahy.20

a Length of the amino acid sequence.

b Molecular weight of the amino acid sequence.

c Isoelectric point of the AhCBLL.

d Number of transmembrane domains, as predicted by the TMHMM Server v2.0.

### Phylogeny, Gene Structure, and Conserved Motifs of the *CBLL* Gene Family in Peanut

**FIGURE 1:**
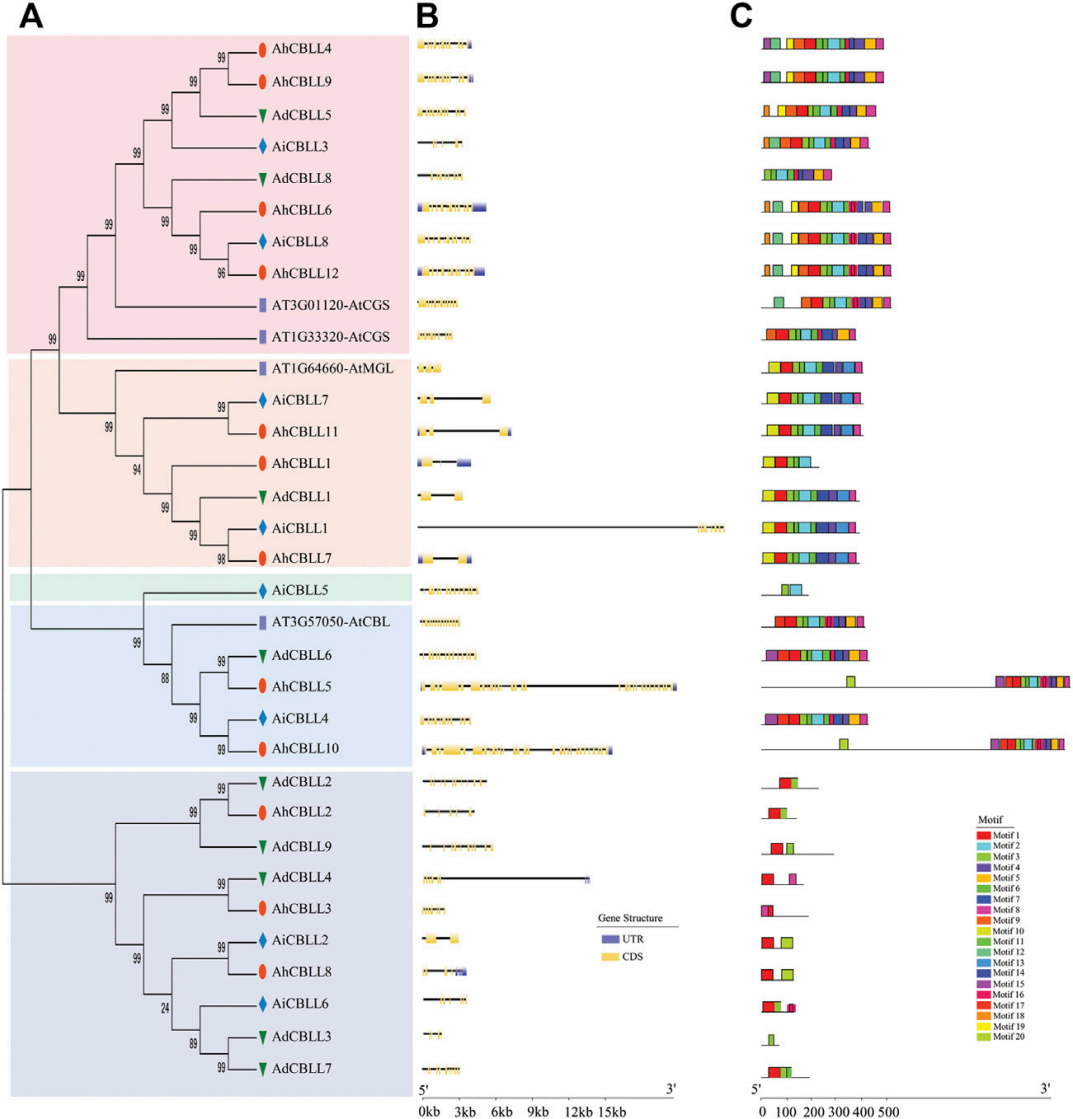
Comparison of the gene structure and motif of 29 *CBLL* genes in peanut. **(A)** Unrooted phylogenetic tree with over 50% bootstrap value above the branch. The clade I, II, III, Ⅳ and Ⅴ were displayed in pink, orange, green, blue and dark blue colors, separately. The names of species were abbreviated to two letters, named as *Arachis duranensis* (Ad), *Arachis ipaensis* (Ai), *Arachis hypogaea* L. *Tifrunner* (Ah). **(B)** Exon/introns and untranslated regions (UTRs) of *CBLL*s. Green boxes denote UTR (untranslated region); yellow boxes denote CDS (coding sequence); black lines denote introns. The length of protein can be estimated using the scale at the bottom. **(C)** Motif architectures of all *CBLL* genes. Each motif is illustrated with a specific color, and the distribution of identified motifs corresponds to their positions.

*Arabidopsis thaliana* emerged is the model organism of choice for in plant biology research, we screened CBLL members through the *A. thaliana* genome, and four members were detected. To investigate the evolutionary relationships of the *CBLL* family genes in peanut, we conducted an NJ-phylogenetic tree, and analyzed the gene exon/intron structural and conserved motifs. The results demonstrated that the 29 peanut and four Arabidopsis CBLL proteins could be integrated into five clades (Figure 1A). Eight, six, one, four, and 10 CBLLs pertained to clade I (CGS clade), clade II (MGL clade), clade III (new clade), clade Ⅳ (CBL clade) and clade Ⅴ (new clade), respectively (Figure 1A). All the four large clades included CBLLs from the cultivated peanut and its two diploid progenitors. Interestingly, these five distinct groups have different gene structure and motif arrangement. The CGS clade had approximately 11 exons, and only *AiCBLL3* had 10 exons. In sharp contrast to those in clade I, the MGL clade possessed two-three exons with one exception (*AiCBLL1*, 10 exon); the CBL clade had the greatest exon number, ranging from 12 to 29. For the two new clades, the only gene in clade III had 13 exons and clade Ⅴ had 2–13 exons (Figure 1B). Further, we identified 20 different conserved motifs (Figure 1C). In general, clade I, clade II, and clade Ⅳ had more motifs than clade III and clade Ⅴ. Motif 1 was the most common, present in all *CBLL* genes except *AdCBLL8*, *AiCBLL5,* and *AhdCBLL3*. Otherwise, the vast majority of CBLLs included motif 2, 3, 4, 6, 8, and 11. Motif 18, and 19 were clade-specific elements in clade I, motif 13 and 14 only existed in clade II, and motif 15 only existed in clade Ⅳ.

### Gene Duplication and Synteny Analyses of Peanut *CBLLs*

**FIGURE 2:**
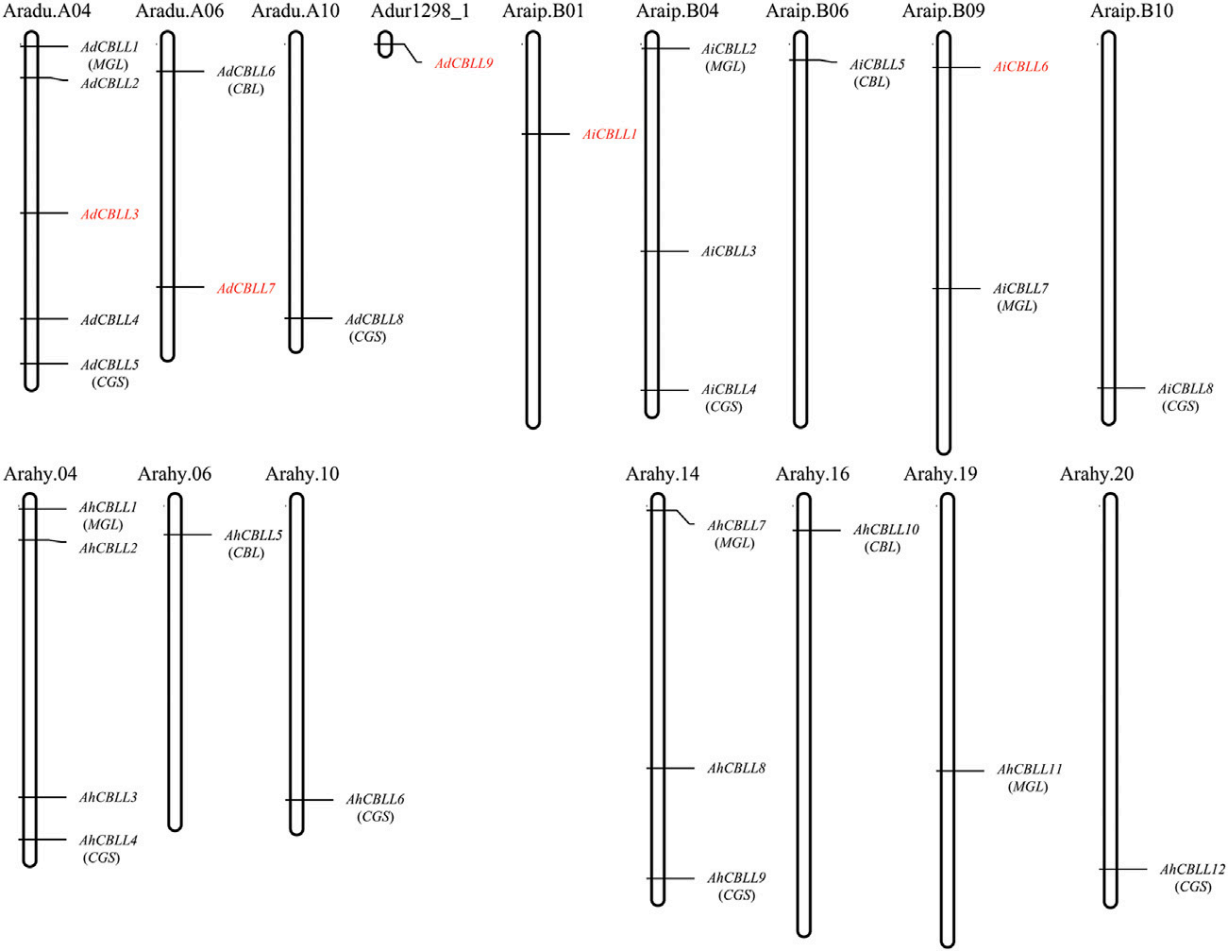
Chromosomal locations of peanut *CBLL* genes. Chromosomal positions of the peanut *CBLL* genes were mapped based on data from PeanutBase. The chromosome number was indicated above each chromosome. Genes in red indicated wild species specific.

**FIGURE 3:**
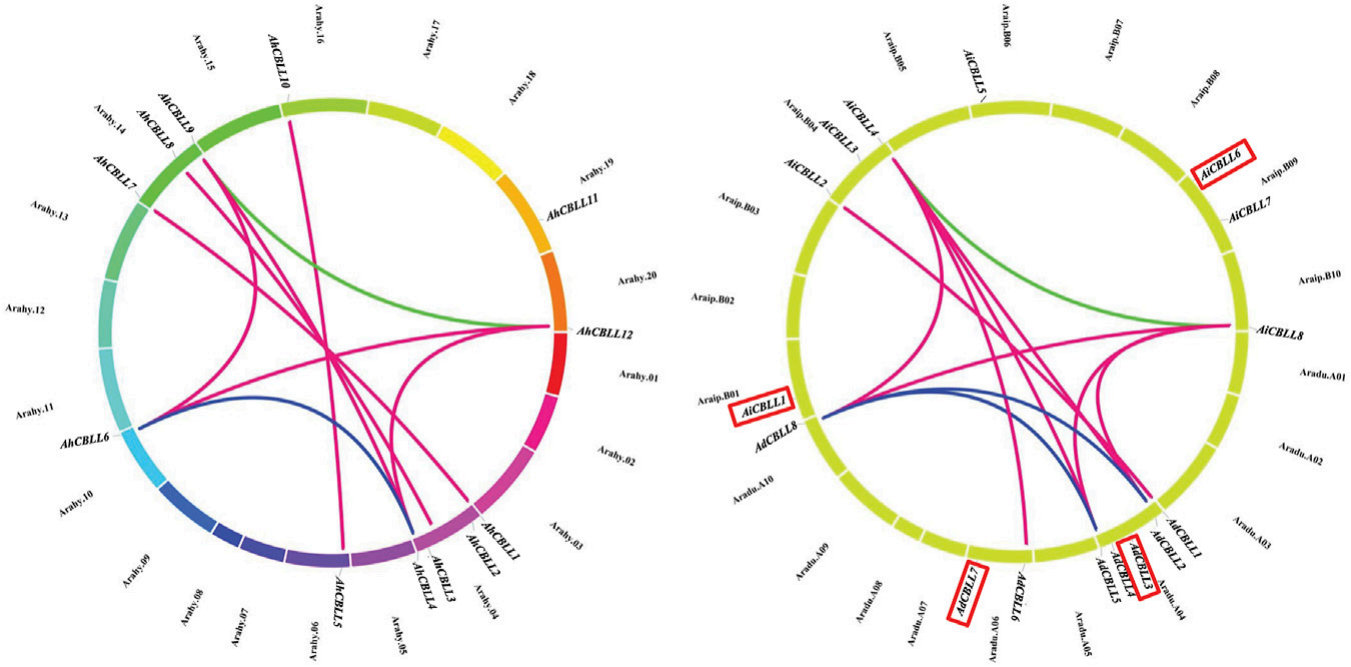
Chromosomal distribution and gene duplications of the *AdCBLL*, *AiCBLL*, and AhCBLL genes. The scales on the circle were in Megabases. Each colored bar represented a chromosome as indicated. Gene IDs were labeled on the basis of their positions on the chromosomes. Red frames indicated wild species specific CBLL genes.

**FIGURE 4:**
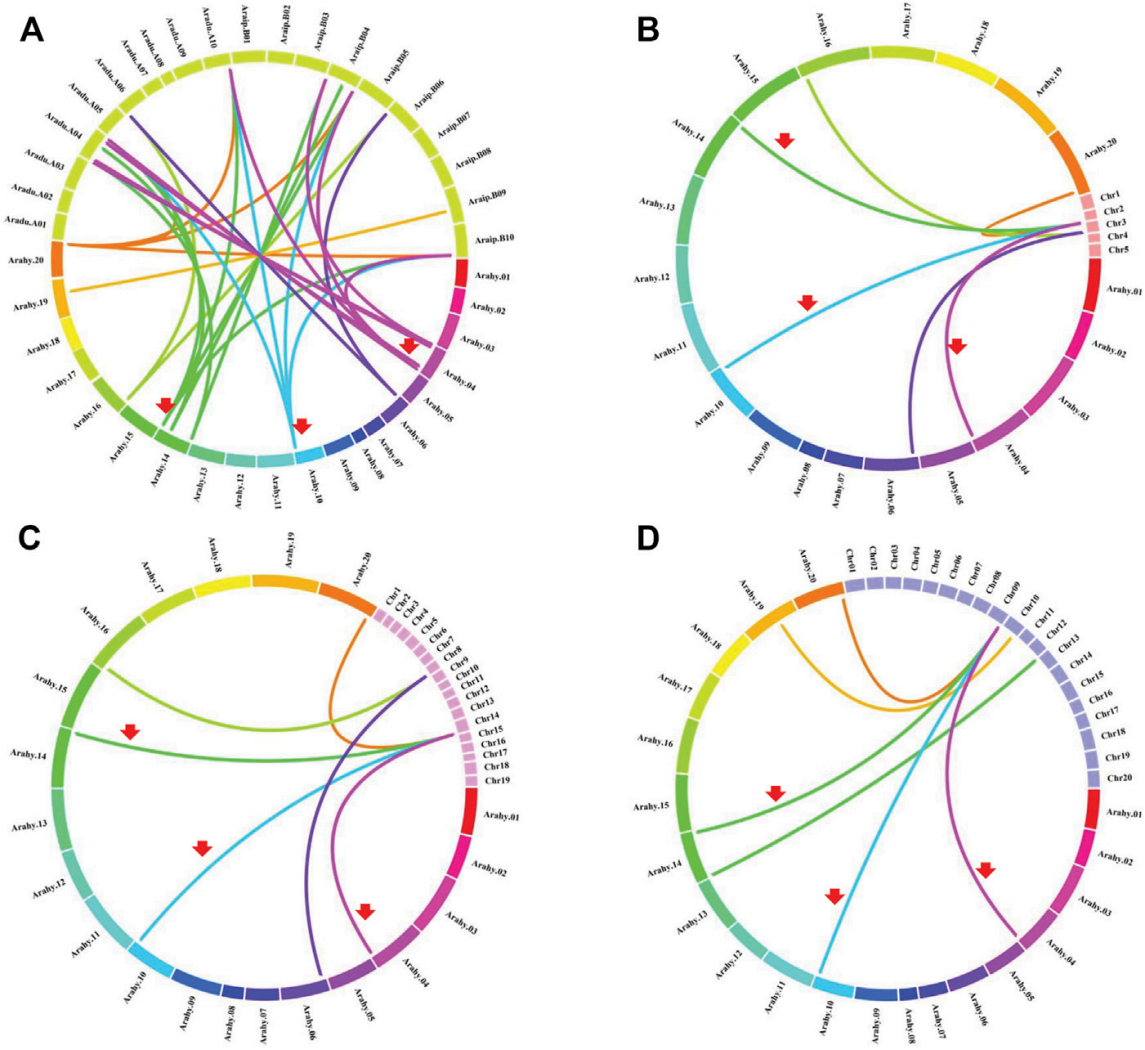
Comparative physical mapping showed the degree of orthologous relationships of *AhCBLL* genes with **(A)** peanut, **(B)** Arabidopsis, **(C)** grape and **(D)** soybean. Red arrows indicated common collinear *CBLL* gene pairs.

Chromosomal location analyses revealed that the 12 *AhCBLLs* were distributed unevenly on seven chromosomes (chromosomes 04, 06, 10, 14, 16, 19 and 20); nine *AdCBLL*s were present on chromosomes A04, A06 and A10; and eight *AiCBLL*s were distributed on chromosomes B01, B04, B06, B09, and B10 (Figure 2). A total of 17 chromosomal fragment repeat gene pairs were identified without tandem repeats (Figure 3 and Supplementary Tables S2–S5). Genomic synteny analyses between cultivated and wild peanut species uncovered five (*AiCBLL1, AiCBLL6, AdCBLL3, AdCBLL7 and AdCBLL9*) wild species-specific *CBLL* members (Figures 2, 3 and Supplementary Tables S2–S5). Further, we calculated the Ks (synonymous) and Ka (non-synonymous) values of the duplicated gene pairs and found that the Ka/Ks ratio for duplicated *AhCBLL* gene pairs ranged from 0.03 to 0.95 with an average of 0.25 (Supplementary Table S6). The ω values of all duplicated gene pairs were less than one, domostrated that purifying selection occurred on these duplicated gene pairs. The whole genome-wide collinear analysis identified that 41.38% (six *AhCBLL* genes, six pairs, Supplementary Table S7; three *AdCBLL* genes, three pairs; three *AiCBLL* genes, three pairs, Supplementary Table S8), 41.38% (six *AhCBLL* genes, eight pairs, Supplementary Table S9; three *AdCBLL* genes, three pairs; three *AiCBLL* genes, three pairs, Supplementary Table S10), and 48.28% (six *AhCBLL* genes, six pairs, Supplementary Table S11; four *AdCBLL* genes, seven pairs; four *AiCBLL* genes, seven pairs, Supplementary Table S12) of the *AhCBLLs* were orthologous with Arabidopsis, grape, and soybean *CBLLs*, but none with rice, respectively (Figure 4). Synteny analysis with soybean, Arabidopsis, and grape revealed three conserved *CBLL* genes (*AhCBLL4, AhCBLL6,* and *AhCBLL9*) in these species. Both collinear and BLAST methods were used to identify *AhCBLL* gene orthologs between peanut and Arabidopsis; 13 orthologous pairs were found (Table 2). The orthologs in Arabidopsis included *AtMGL* participating in Met degradation and plant defense (Ricarda et al., 2005; Goyer et al., 2007; Joshi and Jander, 2009; Atkinson et al., 2013), *AtMOT1/CGS* relating to the regulation of seed growth and metabolism in Arabidopsis (Goto et al., 2002; Hacham et al., 2013; Cohen et al., 2014, 2017; Whitcomb et al., 2018), and *AtCBL* relating to the synthesis of plant hormones (Kim and Leustek., 2000; Levin et al., 2000; Liu et al., 2019). Therefore, we speculated that these *AhCBLL* homologous genes might play multiple roles in peanut growth, development, and stress resistance.

**TABLE 2:** The function of genes homologous to *AhCBLL* genes in Arabidopsis.

Peanut	Arabidopsis	Function	Reference
AdCBLL1/AiCBLL2/AiCBLL7/AhCBLL1/AhCBLL7/AhCBLL11	*AtMGL* (*AT1G64660*)	Regulate methionine degradation, involved in the response to simultaneous biotic and abiotic stresses	Ricarda et al. (2005)
			Goyer et al. (2007)
			Joshi and Jander, (2009)
			Atkinson et al. (2013)
*AdCBLL5/AdCBLL8/AiCBLL4/AhCBLL9*	AtMTO1/AtCGS (AT3G01120)	Influences Met metabolism in seeds, related to light, sucrose and salt and osmotic stresses regulation
			Hacham et al. (2013)
			Cohen et al. (2014)
			Cohen et al. (2017)
			Whitcomb et al. (2018)
*AdCBLL6/AiCBLL5/AhCBLL5*	AtCBL (AT3G57050)	Associated with MET biosynthesis, crucial for embryo patterning and the maintenance of root stem cell niche	Kim and Leustek, 2000
			Levin et al. (2000)
			Liu et al. (2019)

### *Cis*-Acting Element Prediction of Peanut *CBLLs*

**FIGURE 5:**
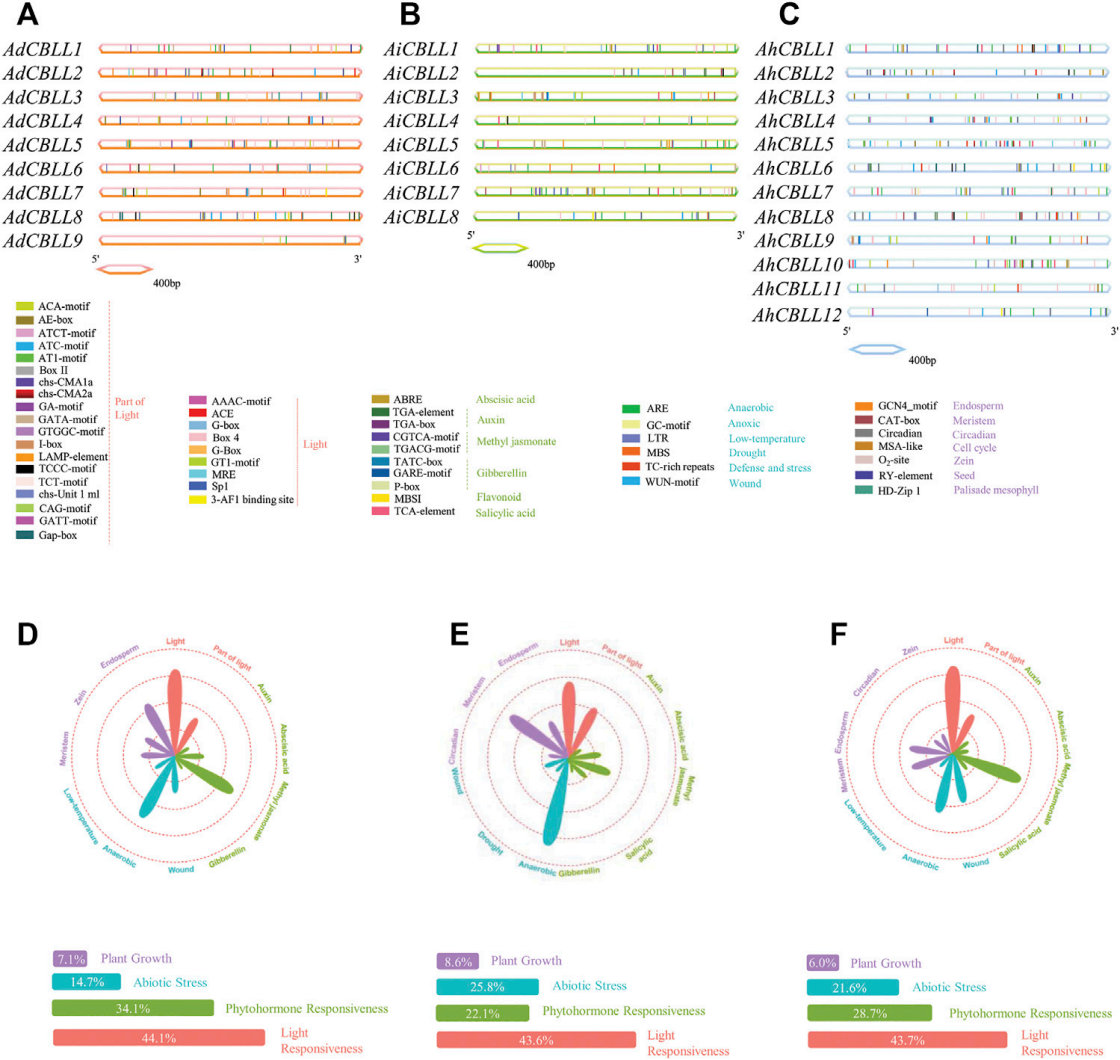
Identification of *cis*-acting elements in all the peanut *CBLL* genes. **(A)** Distribution of *cis*-acting elements in 2 kb upstream of each *AdCBLLs*. **(B)** Distribution of *cis*-acting elements in 2 kb upstream of each *AiCBLLs*. **(C)** Distribution of *cis*-acting elements in 2 kb upstream of each *AhCBLLs*. The different colored boxes indicate distinct promoter elements. **(D)** Assessment *AdCBLL* different subclass and category proportions in a radar chart. **(E)** Assessment *AiCBLL* different subclass and category proportions in a radar chart. **(F)** Assessment *AhCBLL* different subclass and category proportions in a radar chart. The lengths of the petals are proportional to the number of elements in each subclass qualitatively. Purple, blue, green and red petals represent plant growth regulation, abiotic stress responses, phytohormone and light responsiveness, respectively.

We analyzed and predicted *cis*-acting elements in the 2-kb upstream sequences of *CBLL* genes *via* the PlantCARE database. In total, 51 *cis*-regulatory elements were detected (Figures 5A–C), 11 subclasses and four main categories were defined as plant growth, abiotic stress, phytohormone responsiveness, and light responsiveness element groups (Figures 5D–F). In the promoter region of the *AdCBLLs*, the largest subdivision was the light responsiveness group, containing 44.1% predicted *cis*-elements, phytohormone responsiveness elements ranked second (34.1%); abiotic stress response elements were 14.7%, and elements involved in plant growth accounted for 7.1%. *AdCBLL5* had the greatest number of elements with 33 in total (Figure 5D). For *AiCBLLs*, the percentage of light, phytohormone, abiotic stress, and plant growth responsiveness *cis*-elements was 43.6, 25.8, 22.1, and 8.6% (Figure 5E). *AiCBLL7* had the greatest number of elements at 35 in total. In *AhCBLLs*, the similar proportions were 43.7, 28.7, 21.6, and 6.0% (Figure 5F). In the light response category, Box 4tbox4 (light-responsive element) and GT1-motif (part of a module for light response) were the most dominant. Meanwhile, *cis*-acting elements responding to auxin, abscisic acid, gibberellin, flavonoids, methyl jasmonate and salicylic acid were involved in the phytohormone responsiveness group. The CGTCA-motif and TGACG-motif (methyl jasmonate response elements) were followed by ABRE (related to the abscisic acid response). In the abiotic stress response category, ARE (elements regarding oxygen-deficient induction) covered the largest portion, and both WUN-motif (wound-responsive element) and LTR (relating to low-temperature responsiveness) exited in the promoters of *AdCBLLs*, *AiCBLLs*, and *AhCBLLs*. GCN4 motif (elements related to endosperm expression) and CAT-box (referred to meristem expression) were the largest motifs in plant growth regulation. Intriguingly, all types of *cis*-regulatory elements were distributed widely throughout the promoter regions of *CBLL* genes.

### Expression Profile of *AhCBLLs* in Different Tissues of Peanut

**FIGURE 6:**
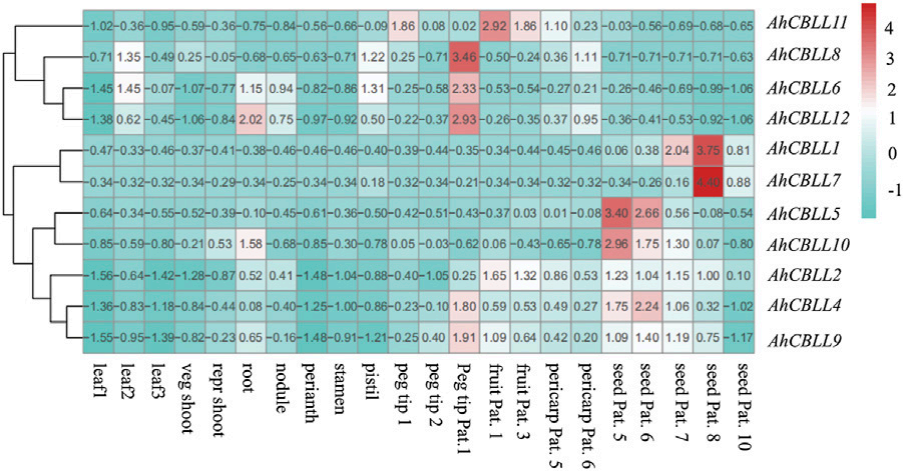
The expression profiles of *AhCBLL* genes. The heat map of the *AhCBLL* gene expression levels was hierarchically clustered using the R package “pheatmap” with the data normalization method of Z-score standardization. The color scale bar ranging from blue to red represents low and high expression, respectively. Abbreviation of the 22 tissues used in the expression profiles of *AhCBLL* genes were as following: seedling leaf 10 days post emergence (leaf 1), main stem leaf (leaf 2), lateral stem leaf (leaf 3), vegetative shoot tip from main stem (veg shoot), reproductive shoot tip from first lateral (repr shoot), 10-day roots (root), 25-day nodules (nodule), perianth, stamen, pistil, aerial gynophore tip (peg tip 1), subterranean peg tip (peg tip 2), Pattee 1 stalk (peg tip Pat. 1), Patte 1 pod (fruit Pat. 1), Pattee 3 pod (fruit Pat.3), Pattee 5 pericarp (pericarp Pat.5), Pattee 6 pericarp (pericarp Pat.6), Pattee 5 seed (seed Pat. 5), Pattee 6 seed (seed Pat. 6), Pattee 7 seed (seed Pat.7), Pattee 8 seed (seed Pat. 8), Pattee 10 seed (seed Pat.10).

Previous studies showed the involvement of *CBLL* family genes during different development stages (Kim and Leustek., 2000; Levin et al., 2000; Maimann et al., 2000; Goto et al., 2002; Joshi and Jander, 2009; Atkinson et al., 2013; Hacham et al., 2013; Song et al., 2013; Cohen et al., 2014, 2017; Whitcomb et al., 2018; Liu et al., 2019). Thus, the holistic expression patterns of peanut *CBLLs* in different tissues are needed to provide more insight into their roles during plant growth and development. Tissue analyses of 12 *CBLL* genes showed distinct tissue-specific expression patterns across the 22 tissues (leaf, stem, root, flower, pod and seed) (Figure 6). *AhCBLL2, 4,* and *9* showed higher expression level in almost all the sink tissues; *AhCBLL5* and *10* were expressed mostly in the early seed developmental stages (seed pattee 5 and 6); *AhCBLL1* and *7* had strong expression during the relatively later seed developmental stages (seed pattee 7, 8, and 10), especially in seed pattee 8. *AhCBLL6* and *12* were highly expressed in root, nodule, and peg tip; Additionally, *AhCBLL6* was enriched in peg tip pat 1 and *AhCBLL11* was expressed highly in the peg and fruit tissues.

### Expression Pattern of the *AhCBLLs* in Plant Hormone Response

**FIGURE 7:**
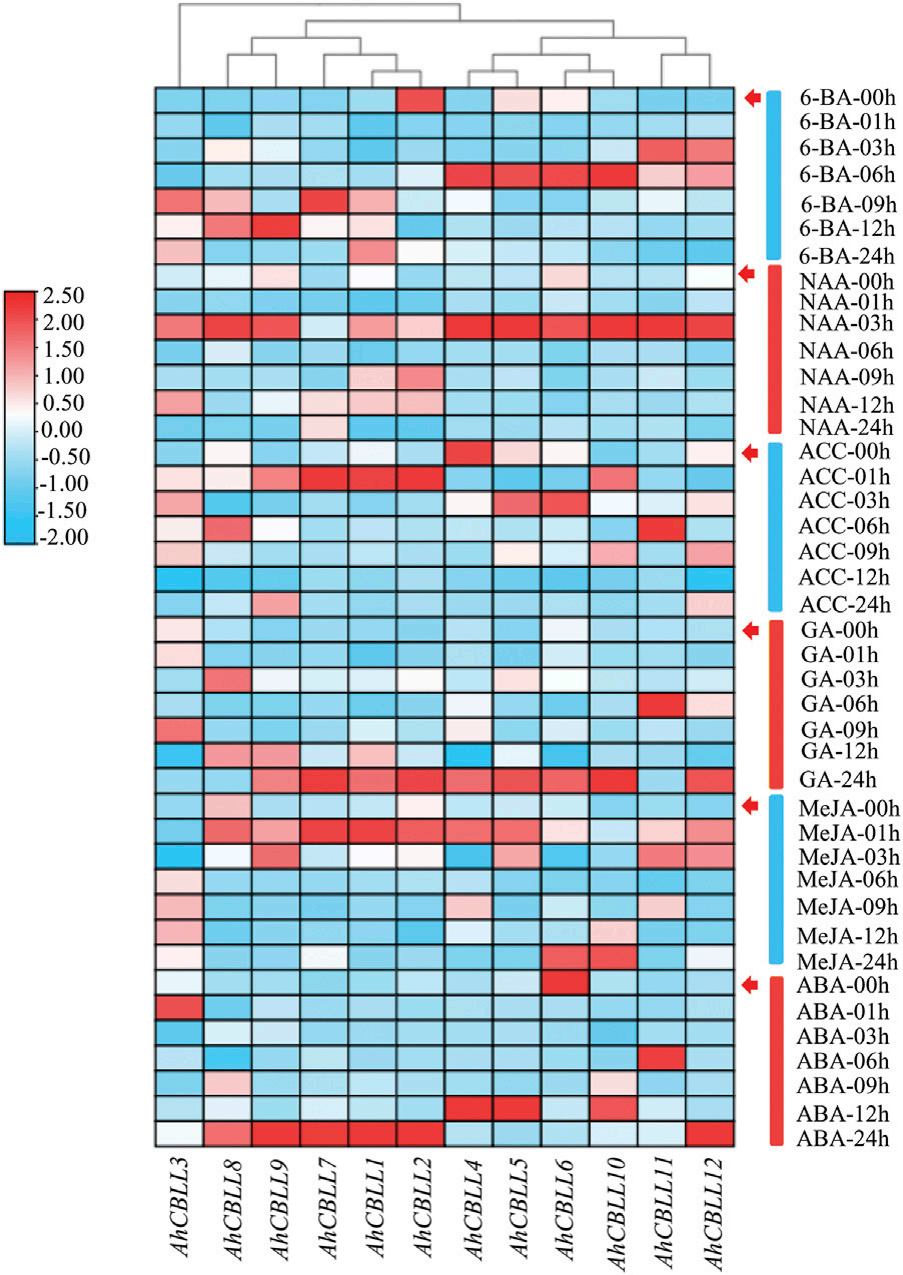
Relative expression of *AhCBLLs* under 6-BA, NAA, ACC, GA, MeJA and ABA treatments. Expression characteristics of *AhCBLLs* in response to different phytohormone at six-time points (00, 01, 03, 06, 09 and 12 h) were normalized to 00 h treatment. The fold changes values were calculated by the 2^−△△Ct^ method and log2 and represented in color scale legend at the left of the heatmap: red indicated up-regulation and blue showed down-regulated expression.

To uncover the possible functions of *AhCBLLs* in response to hormone stress, we conducted qRT-PCR to analyze their relative expressions under 6-BA, NAA, ACC, GA, MeJA, and ABA treatments (Figure 7). In this study, a two-fold change (|log2| > 1) was considered as significantly different for gene expression under each treatment. Of all the 12 *AhCBLL*s, nine genes showed increased expression under all treatments, while the remaining three (*AhCBLL2*, *4* and *6*) had reduced expression at least one treatment (ABA, ACC, or 6-BA). For instance, *AhCBLL6, AhCBLL*4, and *AhCBLL*2 were down-regulated under ABA, ACC, and 6-BA treatments, respectively. Interestingly, the responses of the *AhCBLLs* to these plant hormones were different; *AhCBLLs* responded to NAA, ACC, and MeJA in the early time series, later to 6-BA, and were to slow responded to GA and ABA (mostly at 24 h after treatment). The results indicated that these *AhCBLL* genes might regulate relevant hormone signaling pathways.

### Expression Pattern of the *AhCBLLs* Under Abiotic Stresses

**FIGURE 8:**
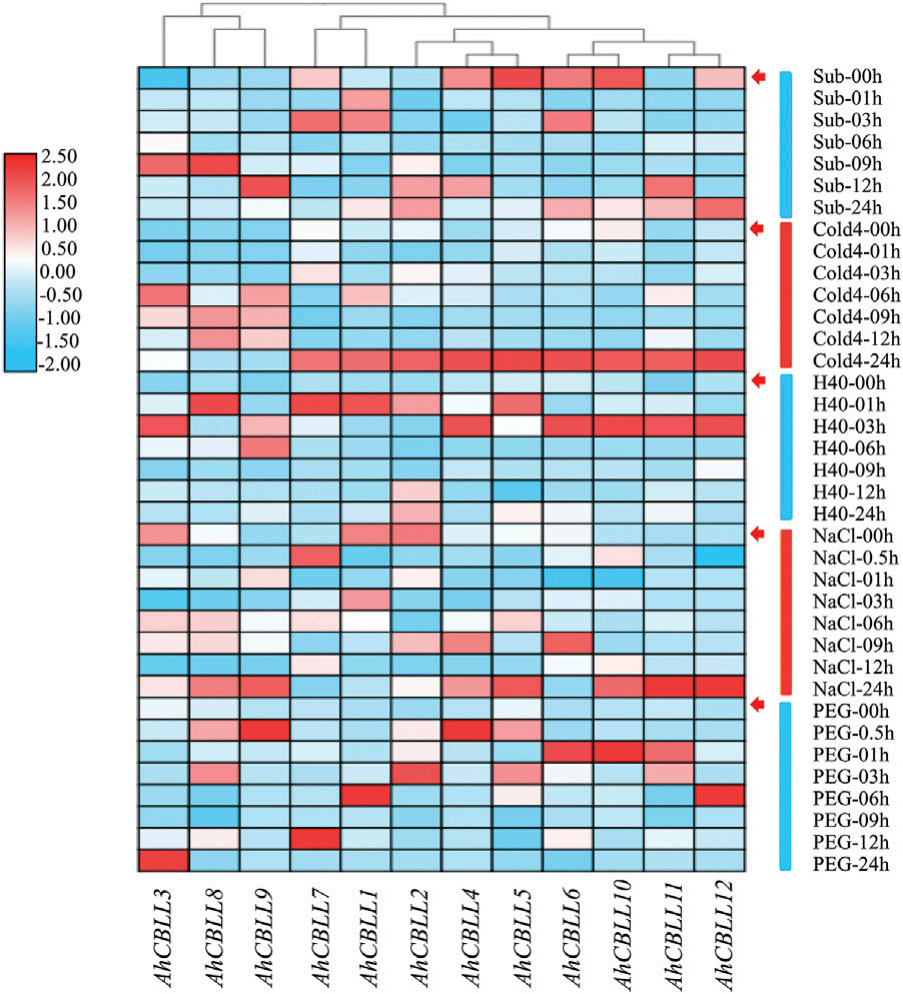
Expression changes of *AhCBLLs* under different abiotic stresses. The abbreviation represented as following, Sub: submergence, Cold4: cold of 4°C, H40: the heat of 40°C, NaCl: 200 mM NaCl, and PEG, polyethylene glycol.

Plants suffer from a wide variety of environmental stressors under natural conditions. We investigated the expression of *AhCBLL* genes, responding to five abiotic stressors (Figure 8). Results showed that the accumulation of *AhCBLL1*, *AhCBLL2*, *AhCBLL3*, *AhCBLL8*, *AhCBLL9,* and *AhCBLL11* transcripts occurred at different time points after the submergence treatment, while the remaining six showed obviously down or up regulated curves. Expression levels of *AhCBLL3*, *AhCBLL8*, and *AhCBLL9* were elevated starting at 6 h under cold stress and the other nine members reached their highest expression at 24 h. Interestingly, all analyzed *AhCBLL* genes showed a positive response to heat stress. *AhCBLL 1*, 5, *7*, and *8* were up-regulated rapidly after 1 h of treatment, while *AhCBLL 3*, *4*, *6*, *10*, *11*, and *12* were strongly upregulated after 3 h of treatment. Moreover, three *AhCBLL* genes, *AhCBLL1*, *-2*, and *3* were inhibited under NaCl stress, and the remaining nine were up-regulated after NaCl treatment at different time points. Surprisingly, all *AhCBLLs* were induced after PEG stress. In summary, *AhCBLL* genes might play important roles in various types of environmental stress regulation.

### Association Analysis of Peanut *CBLLs* with 104 Traits of Peanut

**FIGURE 9:**
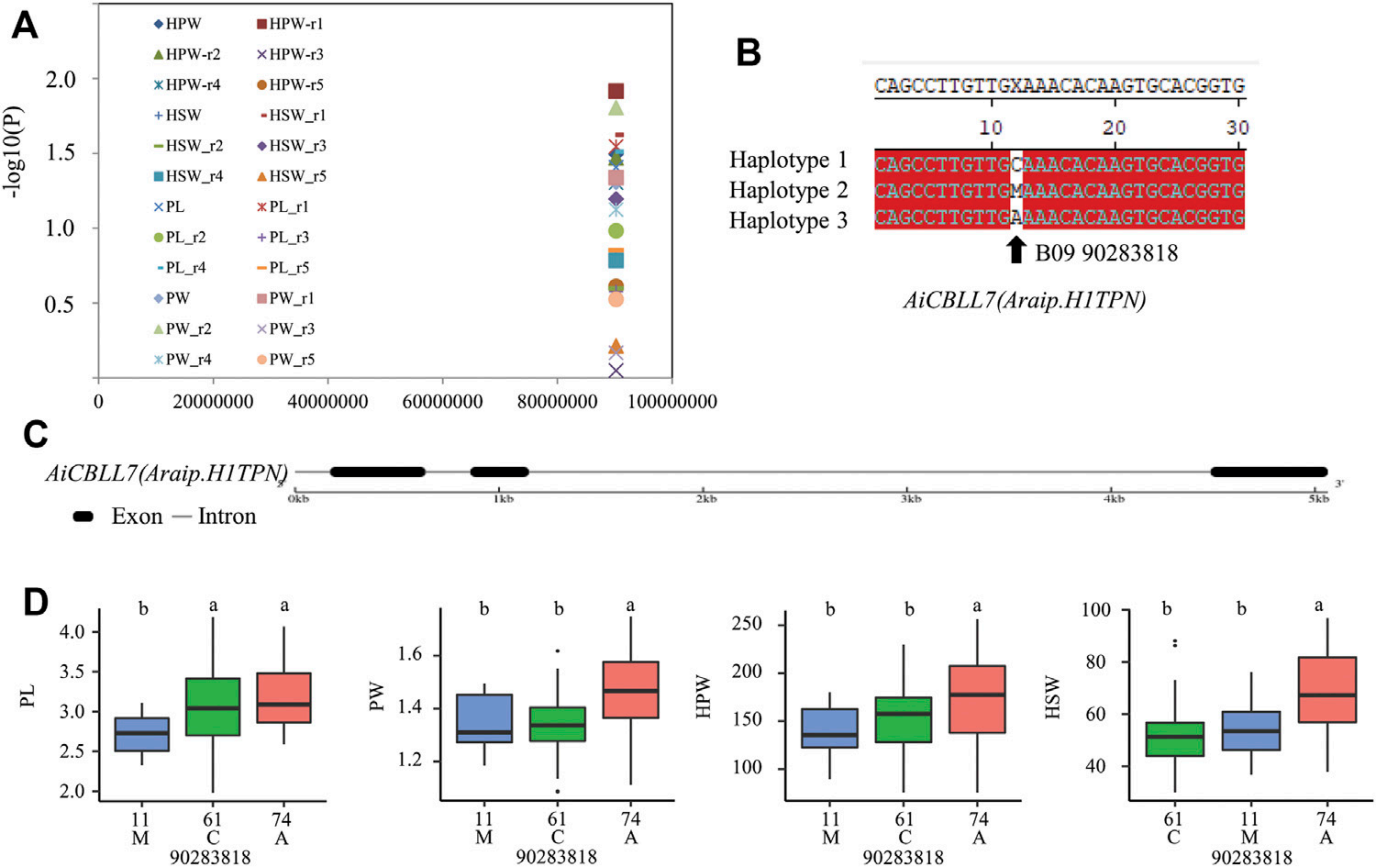
Association mapping results and the phenotypes of the polymorphic sites of the peanut *CBLLs* associated with HPW/HSW/PL/PW variation. **(A)** Association results between HPW/HSW/PL/PW and the polymorphisms in peanut *CBLLs*. **(B)** Sequences of site significantly associated with HPW/HSW/PL/PW variation. **(C)** The gene structures of *AiCBLL7*. **(D)** Phenotypic comparison of haplotypes of the associated site with HPW/HSW/PL/PW in five environments of the population.

To uncover the roles of *CBLL* genes in peanut development and stress response, we performed candidate gene association analysis using five SNPs (single nucleotide polymorphisms) in *AhCBLLs* from transcriptome data of 146 peanut lines, and 104 phenotypes related to peanut development and stress response were collected from the five environments. The results indicated that one polymorphic site [B09_90283818^(C/M/A)^] was significantly associated with PL, PW, HPW, and HSW traits (Figure 9A and Supplementary Tables S13–S15). The site B09_90283818 mainly formed three haplotypes [B09_90283818^(C/M/A)^) (Figure 9B) in the population and were located in the predicted exon region of *AiCBLL7* (Figure 9C). Results showed that PL, PW, HPW, and HSW in haplotype A were significantly higher than those in haplotype C (Figure 9D).

## Discussion

### Duplication Contributed to *AhCBLLs* Expansion

Gene duplication contributes significantly to the proliferation of genes in plant species (Davidson et al., 2013; Hou et al., 2014), and leads to gene diversification or drives the evolution of genes. Based on synteny analyses, nine of the 12 *AhCBLL*s had syntenic relationships, all of them were inherited from A. duranensis and A. ipaensis genomes (Figures 2, 3 and Supplementary Table S2); the main expansion mechanism of *AhCBLLs* was whole-genome duplication (allopolyploidization), while none‐segmental and tandem duplication events occurred in the tetraploid stage.

Duplicated genes often evolve to lose their original functions and/or obtain new functions to enhance the adaptability of plants (Dias et al., 2003). Previous research demonstrates that a diversified expression pattern might be a significant reason for retaining duplicated genes in the genome (Gu et al., 2002). The duplicated genes lead to gene-dose effects or result in functional diversity. We found that all the *AhCBLL* gene pairs from allopolyploid species displayed a similar tissues expression pattern. For example, gene pair *AhCBLL1* (*Arahy.04*)-*AhCBLL7* (*Arahy.14*) was mainly expressed in the later seed development stage. Pat.8; *AhCBLL4* (*Arahy.04*)-*AhCBLL9* (*Arahy.14*) showed a higher expression level in peg. tip. Pat.1, *AhCBLL5* (Arahy.06)–*AhCBLL10* (Arahy.16) were enriched in the earlier seed development stage. Pat.5 and 6, and *Ah CBLL6* (Arahy.10)-*AhCBLL12* (Arahy.20) were highly expressed in root, nodule, and peg tip. Pat.1 (Figure 6). Most of the four allopolyploidy duplicated gene pairs demonstrated similar expression patterns to hormone treatments and abiotic stresses that might result in the way of gene-dose-effect; however, the expression of *AhCBLL4* was inhibited under submergence and ACC treatment while *AhCBLL9* was active ated; *AhCBLL6* was down-regulated under ABA treatment while *AhCBLL12* was up-regulated (Figures 7, 8); this opposite expression profile of the allopolyploidy duplicated gene pairs implied functional diversity. These results suggested that gene duplication of the *CBLL* family in peanut provided intricate regulation of signal transduction.

### Subfunctionalization of the *AhCBLL* Genes in Peanut

The phylogenetic tree divided all the 29 peanut CBLL proteins into four clades (Figure 1A). Eight, six, one, four, and 10 CBLLs pertained to clade I (CGS clade), clade II (MGL clade), clade III (new clade), clade IV (CBL clade), clade V (new clade), respectively. The members in the CGS clade, MGL clade and CBL clade might have similar functions to the corresponding Arabidopsis genes; however, the function of the members in clade III and clade V was uncertain. The annotation of the one gene of clade III and most genes of clade V were CBLs; however, the gene structure and motif arrangement were quite different from those in the CBL clade, indicating divergence in gene function.

The prediction of *cis*-acting elements can provide important clues for the study of gene expression regulation (Zhu et al., 2017). In this study, analysis of the 2-kb upstream sequences of the initial codon of *AhCBLL* genes showed that the *AhCBLLs* contained multiple stress and hormone response elements, but the types and numbers were different. The percentage of *cis*-acting elements to phytohormone in A. duranensis was approximately 12%, which was more than that in A. ipaensis, indicating that the transcriptomic regulation of *AdCBLLs* might be more complex than in *AiCBLLs.* Most *AhCBLL* genes not only had *cis*-acting elements respond to abiotic adversity, but also had elements that responded to hormonal signals such as gibberellin, abscisic acid, salicylic acid and methyl jasmonate (Figure 5). For example, TCA-element, LTR, circadian and TC-rich repeats were exited in the promotor region of *AhCBLL1,* implying that *AhCBLL1* might respond to salicylic acid signals and participate in the regulation of cold stress, circadian rhythm and plant defense response. The positive response of most *AhCBLLs* genes to hormones was the mainstream regardless of the *AhCBL2* and *AhCBL6* were down-regulated in 6-BA and ABA, respectively (Figure 7), implying their relevant roles in these hormone signal pathways*.* Surprising finding was that all analyzed *AhCBLL* genes showed positive response to heat and PEG stresses (Figure 8), suggesting that the *AhCBLL* may be important for peanut resistance to heat stress (Figure 8). Similarly, *AhCBLL1*, *AhCBLL2*, *AhCBLL3*, *AhCBLL8*, *AhCBLL9,* and *AhCBLL11* were found to up-regulated by submergence (Figure 8), indicating the physiological function of these genes in peanut water logging stress tolerance mechanisms. Additinally, the expression of the nine *AhCBLL* genes except *AhCBLL1*, *2*, and *3* were obviously enhanced after NaCl treatment at different time points (Figure 8). Considering the common positive response of *AhCBLL8*, *AhCBLL9,* and *AhCBLL11* under heat, drought, submergence, and salt stresses, overexpression of these three genes in peanut may be an effective method to improve the peanut comprehensive abiotic stress resistance. Many important genes were selectively expressed in specific tissues during various physiological and developmental processes (Wan et al., 2014). Tissue expression of the *AhCBLLs* showed multiple tissue expression patterns suggesting subfunctionalization of this family.

Among the 13 *CBLL* orthologous pairs between peanut and Arabidopsis (Table 2), the functions of the corresponding ortholog genes in Arabidopsis have been determined; they functioned in influencing flowering, cell elongation, pollen tube growth, and played an important role in seed germination (Table 2). *AtMOT1/AtCGS* have been found to affect the physiological and behavioral processes of seeds, related to the biosynthesis of ethylene and polyamines (Goto et al., 2002; Hacham et al., 2013; Cohen et al., 2014, 2017; Whitcomb et al., 2018). Furthermore, *AtMGL*, regulates Met degradation, involved in the response to simultaneous biotic and abiotic stresses (Ricarda et al., 2005; Goyer et al., 2007; Joshi and Jander, 2009; Atkinson et al., 2013). Therefore, these *AhCBLL* orthologous genes may also play multiple roles in peanut development, and plant hormone synthesis or response.

### *AhCBLL* Gene Plays an Important Role in Peanut Pod and Seed Development

The single-nucleotide polymorphic sites in *AiCBLL7* (corresponding to *AhCBLL11*), were significantly associated with PL, PW, HPW and HSW variation. The polymorphic site in *AiCBLL7*, [B09_90283818(C/M/A)], located in the predicted exon region of the gene, B09_90283818(C/M/A) led a 268E to 268 K amino acid transition in the peanut population. These results indicated that B09_90283818(C/M/A) sequence polymorphisms might be the actual functional sites. Further, *AhCBLL11* was mainly expressed in the peg and fruit, especially in early fruit development stages, which provided additional evidence for its function in peanut pod development. Most *AhCBLLs* exhibited tissue-specific expression patterns, almost all the *AhCBLLs* were expressed in higher levels in the peg, fruit, or seed (Figure 6). Further investigation was needed to confirm the roles of *AiCBLL7* (*AhCBLL11*) in the pod and seed development of peanut.

## Conclusion

In summary, this genome-wide identification, characterization and expression analysis of peanut *CBLL* genes provides valuable information for understanding the evolution and molecular functions of the peanut *CBLL* gene family, and highlights potential *CBLL* genes involved in peanut pod (seed) development and abiotic stress responses. The results of this study provide a foundation for further research regarding the function of the peanut *CBLL* gene family.

## Data Availability

The datasets presented in this study can be found in online repositories. The names of the repository/repositories and accession number(s) can be found in the article/Supplementary Material.
