# Supplementary material for: Global Survey, Expressions and Association Analysis of CBLL Genes in Peanut
Source: Front Genet. 2022 Mar 9;13:821163. doi: 10.3389/fgene.2022.821163 (PMC8959419; doi:10.3389/fgene.2022.821163)
Supplement: Supplementary file 9 [file Table6.DOCX]

**|** Ka/Ks calculation of each duplicated *AhCBLL* genes pairs

| **Duplication gene pairs** | **S** | **N** | **Ka** | **Ks** | **Ka/Ks** | **Selection pressure** |
| --- | --- | --- | --- | --- | --- | --- |
| *Aradu.02IGP(AdCBLL1)-*  *Araip.V3B0A(AiCBLL2)* | 300.08 | 977.92 | 0.0062 | 0.0771 | 0.0799 | Purify selection |
| *Aradu.W013I(AdCBLL5)-*  *Aradu.UE7BN(AdCBLL8)* | 219.92 | 704.08 | 0.0471 | 0.5141 | 0.0917 | Purify selection |
| *Aradu.W013I(AdCBLL5)-*  *Araip.26T6F(AiCBLL4)* | 342.58 | 1070.42 | 0.0604 | 0.0863 | 0.6998 | Purify selection |
| *Aradu.W013I(AdCBLL5)-*  *Araip.KUG1C(AiCBLL8)* | 359.50 | 1119.50 | 0.1114 | 0.5853 | 0.1904 | Purify selection |
| *Aradu.JQ7JG(AdCBLL6)-*  *Araip.P8SRT(AiCBLL5)* | 334.50 | 1057.50 | 0.0086 | 0.0090 | 0.9486 | Purify selection |
| *Aradu.UE7BN(AdCBLL8)-*  *Araip.26T6F(AiCBLL4)* | 219.67 | 704.33 | 0.0431 | 0.5119 | 0.0842 | Purify selection |
| *Aradu.UE7BN(AdCBLL8)-*  *Araip.KUG1C(AiCBLL8)* | 220.25 | 703.75 | 0.0014 | 0.0566 | 0.0251 | Purify selection |
| *Araip.26T6F(AiCBLL4)-*  *Araip.KUG1C(AiCBLL8)* | 337.67 | 1066.33 | 0.0718 | 0.5586 | 0.1285 | Purify selection |
| *Arahy.AA870A(AhCBLL1)-*  *Arahy.V9A8GQ(AhCBLL7)* | 183.83 | 566.17 | 0.0261 | 0.1144 | 0.2278 | Purify selection |
| *Arahy.0JH0K6(AhCBLL4)-*  *Arahy.AGM2GS(AhCBLL6)* | 386.67 | 1203.34 | 0.0980 | 0.5707 | 0.1716 | Purify selection |
| *Arahy.0JH0K6(AhCBLL4)-*  *Arahy.I2BVW6(AhCBLL9)* | 387.58 | 1205.42 | 0.0050 | 0.0236 | 0.2117 | Purify selection |
| *Arahy.0JH0K6(AhCBLL4)-*  *Arahy.S7AN2D(AhCBLL12)* | 384.58 | 1196.42 | 0.1005 | 0.5642 | 0.1781 | Purify selection |
| *Arahy.AGM2GS(AhCBLL6)-*  *Arahy.I2BVW6(AhCBLL9)* | 386.58 | 1203.42 | 0.0954 | 0.5690 | 0.1677 | Purify selection |
| *Arahy.AGM2GS(AhCBLL6)-*  *Arahy.S7AN2D(AhCBLL12)* | 405.25 | 1241.75 | 0.0032 | 0.0458 | 0.0705 | Purify selection |
| *Arahy.FW69P6-*  *Arahy.Q4SW2C(AhCBLL8)* | 96.50 | 314.50 | 1.2052 | 3.2433 | 0.3716 | Purify selection |
| *Arahy.I2BVW6(AhCBLL9)-*  *Arahy.S7AN2D(AhCBLL12)* | 384.50 | 1196.50 | 0.0979 | 0.5737 | 0.1707 | Purify selection |
| *Arahy.F5KNV4(AhCBLL5)-*  *Arahy.0AC10P(AhCBLL10)* | 1251.58 | 4193.42 | 0.0067 | 0.0162 | 0.4152 | Purify selection |

S, number of synonymous sites; N, number of non-synonymous sites; Ka, non-synonymous substitution rate; Ks, synonymous substitution.
